# The future of flourishing in veterinary medicine: a systems-informed positive psychology approach in veterinary education

**DOI:** 10.3389/fvets.2024.1484412

**Published:** 2025-01-07

**Authors:** Virginia K. Corrigan, Rebecca L. Newman, Philip Richmond, Elizabeth B. Strand, Josh M. Vaisman

**Affiliations:** ^1^University of Tennessee College of Veterinary Medicine, Department of Academic Affairs, University of Tennessee Institute of Agriculture, Knoxville, TN, United States; ^2^Department of Rural Resilience and Innovation, Veterinary Technology Program, Appalachian State University, Boone, NC, United States; ^3^Flourishing Phoenix Veterinary Consultants, LLC., Odessa, FL, United States; ^4^Department of Large Animal Clinical Sciences, Center for Veterinary Social Work, University of Tennessee College of Veterinary Medicine, Knoxville, TN, United States; ^5^Flourish Veterinary Consulting, Firestone, CO, United States

**Keywords:** positive psychology, wellbeing, veterinary education, positive education, veterinary medicine

## Abstract

Individuals in the veterinary profession are experiencing significant mental health and wellbeing challenges. A holistic view of wellbeing, which encompasses both physical and mental health, underscores their interconnected nature. This integrated approach reduces the artificial separation of wellbeing facets, and highlights how mental states influence not only individuals, but also their interactions with animals, the environment, and others in the workplace. Wellbeing challenges in veterinary medicine may contribute to negative impacts in animal, human, and environmental health. Veterinary education institutions and systems are also experiencing complex challenges as they adapt to rapidly changing societal, workforce, and professional wellbeing related pressures. This review paper explores the field of positive psychology and its application in educational contexts, commonly known as positive education. A thorough exploration of the systems-informed positive education approach and ways in which it can proactively enhance veterinary professional wellbeing from within the veterinary education ecosystem are presented. It is important to recognize that individual self-care, while valuable, cannot compensate for systemic dysfunctions such as poor team dynamics, ineffective leadership, or organizational culture issues. Addressing these systemic factors is critical for creating environments that support sustained flourishing. Positive psychology interventions delivered through the pathways of individuals, groups, and organizations specifically within a veterinary education context are discussed. Limitations, considerations, and proposed measurement strategies are reviewed. The implications of implementing a systems-informed positive psychology approach to enhance wellbeing in veterinary education include creating curriculum and cultures that enable flourishing within veterinary education institutions. Strengthening the individual and collective wellbeing of veterinary professionals has the potential to enhance the quality of care provided to animals, which has myriad positive implications for animal caregivers, their communities, the environment, and society.

## 1 Introduction

Positive psychology is the study of the processes and conditions that contribute to wellbeing and optimal functioning (flourishing) for individuals, groups, and institutions (1). Wellbeing can be simply defined as feeling good and functioning well (2). Wellbeing involves flourishing in multiple areas of life, including physical health and all the pillars of PERMA: Positive emotion, Engagement, Relationships, Meaning, and Accomplishment (3). Positive psychology represents a paradigm shift in psychology, emphasizing the study of positive experiences, traits, and institutions, as opposed to a traditional deficit-based view of the human condition (3).

While this paper focuses on positive interventions and systemic approaches to flourishing, it is essential to recognize the usual focus of addressing poor wellbeing states, such as mitigating stress, reducing burnout, and managing mental health crises. Strategies for these challenges often include employee assistance programs, resilience training, and other reactive measures designed to alleviate existing distress (4, 5). In this review article, an overview of the field of positive psychology will be presented, followed by a brief literature review on positive education (applied positive psychology in education). Next, applications for the implementation of positive psychology in veterinary education will be introduced with evidence-based interventions at the individual, team, and organizational levels, including proposed measurement and assessment strategies. Finally, limitations and considerations will be discussed.

The goal of this literature review is to describe how a systems-informed positive psychology approach may enhance wellbeing and cultivate cultures that enable flourishing in the veterinary education ecosystem. In this paper, the term “veterinary educator” is intended to be holistic and inclusive, and refers to veterinary school faculty (veterinarian, veterinary technician/nurse), house officers (residents and interns), clinical instructors, and any other individual tasked with training future veterinary professionals in a professional environment. It is recognized that veterinary education and clinical teaching occurs both in traditional academic as well as non-academic settings, with increasing numbers of veterinary schools utilizing distributive and non-traditional models of clinical education (6, 7).

## 2 Positive psychology: overview of the field

Dr. Martin E.P. Seligman, a former president of the American Psychological Association, is often credited with founding the field of positive psychology. In his 1998 presidential address, Seligman called for a shift in focus from solely treating mental illness to also promoting mental health and wellbeing (8). This address marked the official beginning of positive psychology as a scientific discipline. Seligman’s PERMA model of wellbeing comprises five interrelated and measurable elements that give rise to individual and collective human flourishing: P- Positive Emotion, E- Engagement, R- Relationships, M- Meaning, and A- Accomplishment (3). A sixth dimension has been added (H-Health), in response to the recognition of the importance of physical health to overall individual wellbeing (9). The newer PERMA +4 model also includes health, in addition to physical work environment, growth mindset, and economic security, as important to work-related wellbeing (10).

Positive emotions both signal and produce flourishing (11), and can enhance interpersonal connection through the increase in prosocial tendencies as well as through the phenomenon of “positivity resonance” (12). Engagement refers to the deep psychological connection and involvement individuals experience when they are fully absorbed in activities, often termed “flow” (13). Relationships are frequently considered the most important aspect of PERMA, even called the “royal road to wellbeing” (3, 14). Strong positive relationships are associated with a longer life, improved immune functioning, more rapid recovery from illness, and reduced risk for anxiety and depression (15). Meaning refers to the sense of purpose and direction that arises from serving something larger than oneself, and emphasizes the importance of having a clear understanding of one’s core values and goals, and how these align with contributing to the greater good (16). Accomplishment refers to the pursuit and attainment of goals that bring a sense of achievement and success (3).

Positive psychology has undergone three significant waves of research development, each expanding the field’s scope and depth. The first wave of positive psychology research focused on hedonic wellbeing, which is the study of pleasure, happiness, and life satisfaction (17). Researchers in this wave explored subjective wellbeing, examining how people evaluate their lives and what factors contribute to happiness (18, 19). The second wave introduced the concept of eudaimonic wellbeing, which goes beyond pleasure to consider meaning, purpose, and self-realization (17). The origins of the word eudaimonia date back to Aristotle, when the term referred to a state or condition of ‘good spirit’, and the highest human good (20). The term has deep roots in the concepts of positive human virtues and character strengths (20).

The third and most recent wave of positive psychology research adopts a more integrative and contextual approach, both broadening the field and recognizing the ever-increasing complexity of human wellbeing (17). The third wave moves beyond the individual to explore groups, organizations, and systems, with considerations of how context, culture, and individual differences shape wellbeing (17). A deeper and richer exploration of the environmental context of flourishing includes the study of how ecological and interpersonal factors can contribute to the creation of nurturing environments, including an expansion on research methodologies used to better understand these complexities (17). The three main settings where this broader contextual approach has been researched thus far includes workplaces, families, and schools (17). A new paradigm of Systems Informed Positive Psychology (SIPP) is emerging, which incorporates principles from systems science to enable both individuals and social systems to flourish and thrive (21).

Today, positive psychology continues to evolve, with numerous state-of-the-art discoveries and practical applications. One significant advancement is in the field of positive neuroscience, which investigates the brain mechanisms underlying positive emotions and traits, and how brain networks that implement positive emotions are both flexible and modifiable (22). Studies using functional MRI have demonstrated structural plasticity of the brain in response to socio-affective and cognitive mental training, along with correlates associated with hedonic and eudaimonic wellbeing, providing a biological basis for positive psychology interventions (23, 24). The neural basis of life satisfaction and wellbeing are also being explored (25).

Positive psychology interventions (PPIs) are defined as interventions with the goal of enhancing wellbeing that are achieved through positive psychology theory-based pathways (26). Positive psychology interventions have been developed and evaluated for their effectiveness in enhancing individual and collective wellbeing across diverse populations (27, 28). Individual-level PPIs have been shown to increase wellbeing, quality of life, and strengths use; and decrease anxiety, depression, and stress (27). PPIs have also been explored in organizational settings. PPIs applied in organizational settings include job crafting (29), strengths-based coaching (30), appreciative inquiry (31), and positive leadership (32). Workplace wellbeing has recently been strongly linked to company performance (33), proving a compelling business case for the contemporary importance of positive psychology and efforts to integrate PPIs.

Globally, positive psychology influences education, public policy, and sustainable development programs. Positive psychology-based education initiatives have been shown to improve both student wellbeing and academic performance across cultures and contexts (34, 35). The World Happiness Report, which ranks countries based on their citizens’ wellbeing, uses positive psychology principles to measure subjective wellbeing and has been utilized to make policy decisions (36). Additionally, initiatives like the United Nations’ Sustainable Development Goals emphasize good health and wellbeing as a critical component of sustainable development (37). The Inner Development Goals are designed to guide human beings in the inner and collaborative skills that will be needed to accomplish the Sustainable Development Goals (38).

As the field of positive psychology progresses, the application from a contextual and systems-informed approach can contribute to collaborative approaches to enable individuals, communities, and nations to move toward higher levels of flourishing (17). Digital platforms and artificial intelligence applications provide additional frontiers and offer innovative methods to deliver positive psychology interventions in a variety of contexts and in a personalized manner (39–41). With climate change and other complex challenges at the forefront of the global agenda, regenerative positive psychology is being proposed as a new science of wellbeing to reorient the field toward expanding and protecting the health of the life-sustaining systems necessary for wellbeing (42). This overview of positive psychology sets the stage for exploring its applications in various educational contexts, laying the groundwork for the following discussions on systems-informed approaches to flourishing.

## 3 Wellbeing in the veterinary profession

Upon graduation from veterinary school, new professionals take the Veterinarian’s Oath (43). The oath states, “Being admitted to the profession of veterinary medicine, I solemnly swear to use my scientific knowledge and skills for the benefit of society through the protection of animal health and welfare, the prevention and relief of animal suffering, the conservation of animal resources, the promotion of public health, and the advancement of medical knowledge. I will practice my profession conscientiously, with dignity, and in keeping with the principles of veterinary medical ethics. I accept as a lifelong obligation the continual improvement of my professional knowledge and competence” (“Veterinarian’s Oath” 2024). The Veterinary Technician oath is similar in language (44).

The recent 2017 revision of the Declaration of Geneva, which is the modern physician counterpart to the Hippocratic Oath, now includes the pledge: “I will attend to my own health, wellbeing, and abilities in order to provide care of the highest standard” (45). This clause was added based on recommendations from the recently adopted World Medical Association Statement on Physician Well-Being (46). Recent research has expanded upon the connection between increased physician wellbeing and improved patient care (47) and patient-provider communication (48). It has recently been suggested that veterinary medicine would benefit from adopting a stance affirming the importance of provider wellbeing in its own professional oath (49). Whether in the Oath or elsewhere, the separation of health and wellbeing is a persistent theme. Emphasizing flourishing—a state beyond mere survival, characterized by optimal health and functioning—can help articulate the goal of achieving a thriving state for individuals, animals, and the systems they inhabit.

The veterinary profession is facing significant systemic wellbeing challenges. Veterinary medicine is a career path that is often chosen very early on in life, with an interest and high intrinsic value for animals usually involved in the decision-making process (50–52). For example, a growing shortage of veterinary professionals illustrates the systemic pressures contributing to wellbeing challenges. The contemporary realities of the profession include higher levels of mental health and wellbeing challenges than the general population, alongside high levels of turnover, attrition, and burnout in the veterinary workforce (4, 5, 53). A growing shortage of veterinary healthcare professionals leads to ethical and public health dilemmas surrounding lack of access to care for animals (54). Furthermore, the cost of veterinary care is rising, making accessible care even harder to come by for many families and animal caregivers (55). This leads to unnecessary animal suffering and increased moral distress on behalf of the animal healthcare providers (56).

Veterinary professionals, who are typically dedicated and altruistic professionals, often face unsustainable working conditions (49). These conditions have been normalized within the medical professions, contributing to a culture that perpetuates and glorifies excessive stress, long hours, and sleep deprivation (49, 57). In veterinary education, trainees (students, residents, and interns) have been found to experience a poor work-life balance, high levels of burnout, and low levels of mental health (58, 59). High rates of depression and anxiety have been reported in veterinary students (60, 61). Poor physical health and lower levels of physical activity were consistent predictors of poor mental health in veterinary students (60, 62). On average, veterinary students have been found to sleep less than 7 h at night and to exercise only twice weekly (62). A recent systematic review revealed that the prevalence of mental health issues in veterinary students is significantly higher than in other health profession student populations (59). Similar wellbeing challenges are also significantly impacting veterinary educators, with the additional factor of workforce shortages of veterinarians in academia (59, 63, 64). These challenges likely intersect and exacerbate each other, with more research needed to inform integrated solutions.

Additional wellbeing challenges are faced by veterinary professionals due to the inherently challenging moral and ethical dilemmas in veterinary work (65). Moral distress is characterized by psychological disequilibrium, painful feelings, and barriers that present to actions and behaviors aligned with one’s conscience (56, 66, 67). In veterinary practice, moral distress arises from conflicts between the veterinary professional’s responsibilities toward animal welfare and the financial and emotional interests of animal caregivers and clients (65). Examples include providing futile care (provision of care that the veterinarian does not deem necessary), the influence of finances and profits in the business of providing veterinary care, and euthanasia (68). High societal pressure, challenging working conditions, and lack of proper training in ethical conflicts and ethical decision-making can contribute to mental health issues, along with burnout and lower levels of professional fulfillment (56, 65).

The wellbeing challenges in veterinary professionals negatively impacts their ability to provide essential animal healthcare and public health services (4). The veterinary profession is integral to One Health, a concept that refers to attaining optimal health for people, animals, and the environment (69). A recent cross-sectional veterinary industry study with over 14,000 responses found that 30% of the veterinary professionals surveyed planned to leave their current positions, and of those individuals, 50% planned to leave clinical veterinary practice entirely (70). The authors of the report suggested that developing sustainable strategies to retain individuals in the profession is paramount (70).

Proactive strategies to increase veterinary professional wellbeing are becoming evident across the profession. Veterinary social work is a growing interprofessional field which provides services at the intersection of social work and veterinary practice (71). One of the four pillars of veterinary social work is “intentional wellbeing,” which refers to all the elements that impact the wellbeing of animal related professionals, including the purposeful action of building healthy individuals and systems (72). In the recently reported World Small Animal Veterinary Association (WSAVA) wellness guidelines for veterinary practitioners, the importance of addressing wellbeing proactively at the individual, team, and organizational and environmental levels is addressed (4). There is a growing interest in systemic level wellbeing initiatives within the veterinary education field (73, 74).

## 4 Positive education: a brief literature review

Positive education integrates the principles of positive psychology with traditional educational practices to foster both academic success and wellbeing (75). Positive education, as a concept, was significantly influenced by the principles of positive psychology (75). Positive education has been defined in multiple ways, reflecting its multifaceted nature. Seligman et al. (75) describe positive education as education for both happiness and traditional skills. Positive education has also been defined as a methodology that aligns best practice teaching with the science of positive psychology to support and encourage flourishing for individuals within the community and the educational institutions (76).

Demands on educational systems have risen in response to increased global challenges and considerations around our modern students growing up in an increasingly volatile, uncertain, complex, and ambiguous world (77). Positive education can be considered as both a discipline and a perspective emerging from and aligning with the research and application of positive psychology (78). The importance of learning institutions on placing wellbeing and flourishing at the heart of educational missions has been highlighted in the response to the COVID-19 pandemic (78) and the increased urgency to address mental health and wellbeing, particularly for younger generations (79, 80). Core ingredients of positive education (i.e., the skills being developed) include understanding of basic psychological needs and other constructs central to the study of positive psychology and human flourishing (81).

The scope of positive education has widened in recent years from a focus on individuals to a systems-informed approach (35). Educational spaces can be considered as complex ecosystems with many elements (82). In systems thinking, a central maxim is that everything is connected, and one element of the system cannot be optimized at the expense of the entire system (83). The educational ecosystem includes the important populations of students and educators, as well as non-teaching staff, administrators, families, parents, curriculum, legislation, policy, and funding considerations, which are all interrelated and interdependent (82). Sensing and seeing the system as a whole is an important lens for a systems informed positive education (SIPE) perspective (82). The SIPE approach advocates for embedding wellbeing within the fabric of educational institutions in order to achieve lasting and sustainable positive impacts (82).

The implementation of positive education involves various implementation strategies and frameworks. A notable example is the whole-school approach adopted by Geelong Grammar School in Australia (84). This model emphasizes learning, living, teaching, and embedding positive psychology principles within the school environment (84). For instance, encouraging reflection through structured journaling activities, mindfulness exercises integrated into daily routines, and facilitated discussions around character strengths are practical ways to embed these principles into education. A variety of approaches to positive education exist, including (but not limited to) character strengths education and interventions, growth mindset, resilience, coaching and mentoring, mindfulness, wellbeing competencies, and social and emotional learning (78). Individual, group, and organizational level implementation is important (85) as an over-emphasis on individual attributes and skill development may negate the impact of external experiences, cultural contexts, and broader systemic pressures (81).

Numerous factors contribute to the success and sustainable implementation of positive education initiatives (34). These include the involvement of all school stakeholders and the integration of positive education principles into the school’s mission and curriculum, as well as prioritizing educator and community wellbeing (86). Significant challenges to educator wellbeing include heavy workload, time constraints, and the feeling of being undervalued or unappreciated (86). Educator training and ongoing professional development to enhance wellbeing and other professional competencies are crucial, as they ensure that educators can effectively both model and deliver positive education (86).

Positive education has been found to have impacts on both student wellbeing and academic success. Positive education has been found to significantly decrease anxiety and foster positive relationships among students, teachers, and parents (84). Studies from Hong Kong illustrated the impact of positive education at different educational levels, from primary schools to universities, which found that positive education effectively reduced students’ negative emotions and anxiety, increased their motivation and positive personality traits, and improved teacher-student relationships and family dynamics (87). A recent meta-analysis examined the relationship between student wellbeing and their academic achievement involving over 50,000 student participants (88). The study revealed a significant positive correlation between general wellbeing and academic performance, providing evidence to support holistic approaches to wellbeing in educational settings (88).

Positive education approaches have also been found to impact educator wellbeing. Studies have shown that faculty and staff who exhibit higher levels of wellbeing tend to experience greater job satisfaction, enhanced creativity, and improved performance (89). Recent research in Australia revealed that educator wellbeing was most strongly influenced by whether the educational institution prioritized wellbeing, the perceived level of autonomy over the work performed, and the support of leadership and management (86). High educator wellbeing was correlated with many desirable outcomes, including improved educator retention, health and sleep quality, positive relationships with students, and student engagement and academic outcomes (90).

A recent meta-analysis evaluating educator wellbeing highlighted several additional key findings (91). The paper systematically examined 173 independent studies encompassing over 89,000 participants to identify the most important factors associated with educator wellbeing (91). The strongest positive predictors of overall wellbeing were autonomy, hope, competence, and strong interpersonal relationships (91), all constructs central to positive psychology (3). Educators with higher levels of wellbeing were less likely to be experiencing burnout and job turnover, and more likely to be engaged and committed to their job (91).

## 5 Positive psychology, wellbeing, and veterinary education: opportunities and challenges

The consideration of how to enhance wellbeing within the veterinary educational ecosystem is complex, as the field is currently undergoing seismic shifts and disruptive transformation (92). The concept of flourishing, central to positive psychology, is quite relevant in the contemporary context of veterinary education. Veterinary educational institutions in the United States are currently experiencing significant challenges, including the recruitment and retention of veterinary educators, who are crucial to the future of the profession (64). A systems-informed multifaceted collaborative approach to strengthen the veterinary educator workforce and enhance wellbeing in veterinary education has been advocated (64). A systems-informed approach to positive psychology and positive education have similarly been proposed to create sustainable positive change in large and complex systems (21, 82).

### 5.1 Systems-informed positive veterinary education

**Figure 1:**
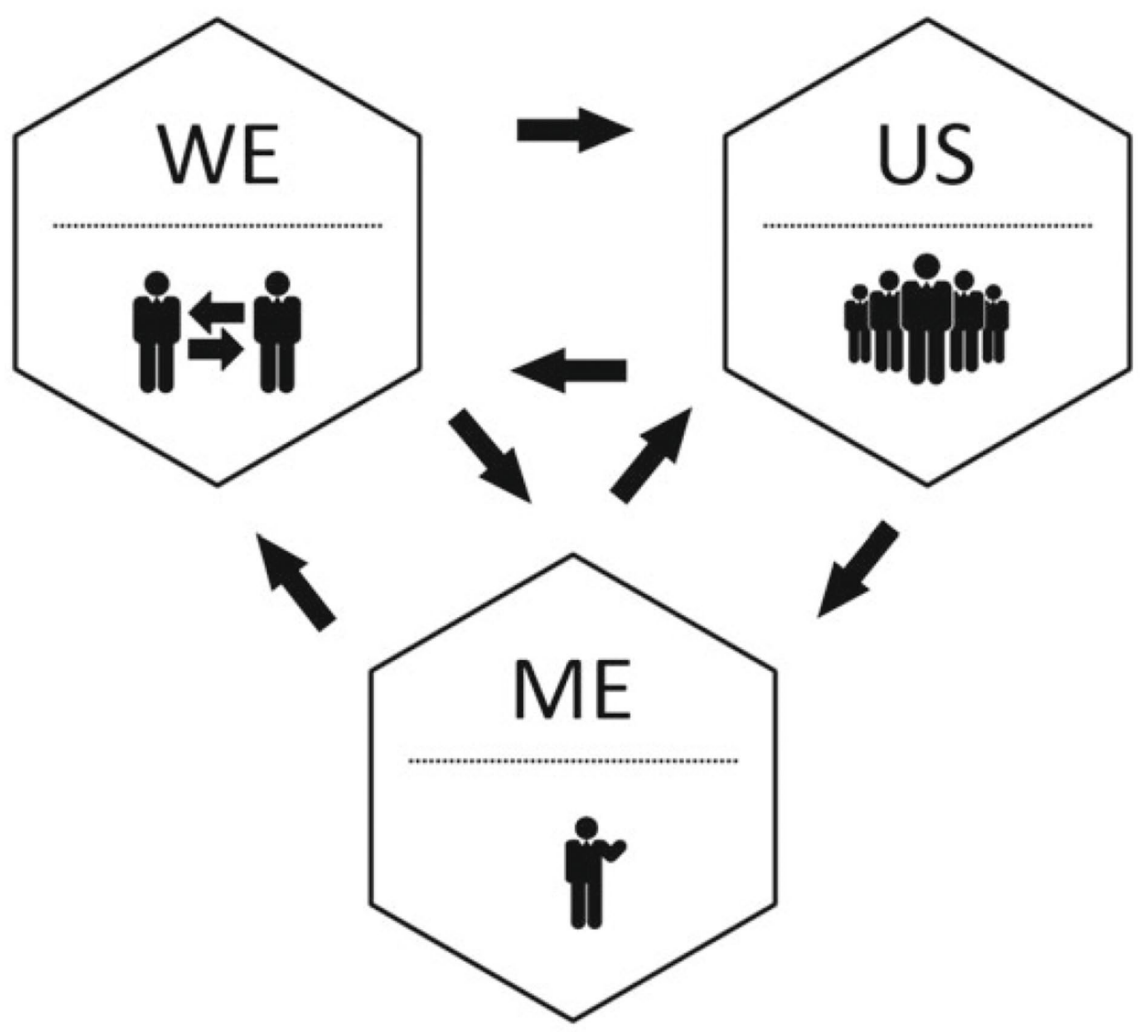
Me, we, and us levels of wellbeing in a system (242).

Positive psychology interventions (PPIs) are evidence-based, intentional strategic enhancements that apply the principles of positive psychology to improve wellbeing by enhancing that which causes and/or contributes to flourishing (27). PPIs have been shown to have both short and long-term positive effects on wellbeing (27). Evidence-based PPIs relevant to veterinary education are presented at the individual, group, and organizational levels, to align with the SIPE approach to positive education (35, 85). Within an educational community, wellbeing interventions and programs can happen at three distinct levels: the individual level (me; to include students, educators, school administrators, parents, etc.), the group level (we; to include pairs or teams of students, educators, classes, departments, etc.), and the organizational level (us; to include the educational institution as a whole community; Figure 1) (85).

#### 5.1.1 Individual-level (me) interventions

Individual-level wellbeing interventions include tasks and strategies that educators and students can do on their own, such as strengths assessments and mindfulness programs (14, 85). Most of the research on individual-level wellbeing in veterinary education has focused on veterinary professional students, interns, and residents (58, 59, 62, 74). Integrating mental health support and wellbeing education into the veterinary curriculum has been recommended to provide ongoing benefits and better prepare students for the emotional demands of the veterinary profession (59). The World Small Animal Veterinary Association (WSAVA) professional wellness guidelines provided further details (4). Individual-level programs that were deemed most important by the WSAVA included healthy lifestyle, wellbeing self-assessment, self-care resources, training in wellness strategies and resilience, and dealing with challenging situations such as compassion fatigue and conflict management (4). The American Association of Veterinary Medical Colleges (AAVMC) Wellbeing Committee is currently developing individual wellbeing competencies to align with competency-based education frameworks (73).

Despite a pressing need, much less attention has been spent on studying wellbeing among veterinary faculty (63, 64, 93). Individual-level PPIs presented in the following section will be holistically oriented to both veterinary students and veterinary educators, which include the exploration of wellbeing competencies, character strengths interventions, and physical activity and mindfulness practices.

##### 5.1.1.1 Wellbeing competencies

A recent systematic review highlighted the need for education that prepares veterinary professionals to handle ethical dilemmas and moral stressors, more effectively communicate with staff members and clients, incorporate work-life integration habits, and to understand and promote team and individual resources (94). The current AAVMC Competency-Based Veterinary Education (CBVE) framework includes “attending to wellbeing of self and others” as a core competency (95). Individual-level positive psychology concepts and skills that could be integrated to develop wellbeing competencies include wellbeing literacy (96), resilience (75, 97, 98), character strengths (99), hope and goal-directed energy (100), optimism (101), active constructive responding (102, 103), growth mindset (104), reflective practices (105, 106) and performance psychology (107). Additional individual-level PPIs include savoring (108), “three good things” (109), and other forms of gratitude practice (110). Understanding basic human psychological and physiological needs would also be a fundamental wellbeing competency, including Maslow’s Hierarchy of Needs (111), with an emphasis on the importance of safety, appreciation, and belonging when working in healthcare settings (70, 112).

Another essential wellbeing competency for veterinary professionals is lowering the stigma associated with proactive help-seeking behavior (113). Individuals attracted to a career in veterinary medicine expect to be fulfilled by applying valuable skills to help animals and to be appreciated by grateful animal caregivers (114). Often veterinary professionals have idealistic and perfectionistic traits that may not be well suited to client expectations, the realities of providing veterinary care, and the costs of veterinary services (115). Overcoming potential stigma associated with proactively pursuing mental health and wellbeing support is essential (116). Despite the fact that veterinary professionals exhibit higher reported rates of mental ill-health than the general population, veterinary professionals have lower reported rates of help-seeking behaviors (117, 118). In a recent study, among those with serious psychological distress, 59% of veterinarians and 65% of veterinary support staff responded that they needed mental health treatment in the past year but did not get it (5). In recent studies of veterinary students in the U.S. and Australia, improving coping strategies and lessening self-stigma, which is related to self-efficacy, are proposed as pathways to increase proactive help-seeking behavior (119, 120). Fortunately, the overall belief that mental health care can help has been increasing, based on serial findings from the series of Merck Veterinary Wellbeing Studies from 2017 to 2022 (5).

##### 5.1.1.2 Character strengths interventions

Character strengths interventions are gaining momentum in applied positive psychology practice to enhance wellbeing within educational systems (121). Character strengths interventions are based on the VIA classification of character strengths (14), which identifies 24 strengths that are valued across cultures and contexts, and contribute to individual and collective wellbeing (20, 122). The 24 character strengths are categorized into six universally recognized virtues: wisdom, courage, humanity, justice, temperance, and transcendence (14). Signature strengths are the most central character strengths to an individual’s identity, which are most energizing to use and most natural to express (122). The foundational idea is that character strengths are positive traits that can be developed, and serve as pathways to flourishing and the elements of PERMA (14). Character strengths involve a process of self-actualization as well as enhancing outcomes for others and contributing to the greater good (14).

Character strengths interventions promote a strengths-based approach to wellbeing using one of the most widely used and highly regarded tools in the field of positive psychology (121). Interventions that focus on character strengths have been shown to improve wellbeing, increase happiness, and enhance connection and resilience (99). In a cross- cultural meta-analysis, using signature strengths in new ways led to both short and long-term increases in happiness, higher levels of flourishing, higher strengths use, and less depression (123). In educational settings, integrating character strengths interventions has been found to help create a common language that can support a positive and collaborative environment and enhance connection between students and educators (121).

##### 5.1.1.3 Physical activity and mindfulness practices

Physical health is essential for maintaining overall wellbeing. Evidence-based physical activity guidelines for adults have been developed by the U.S Department of Health and Human Services (HHS) (124). The health benefits of abiding by exercise recommendations are well established, which include improvement in immunity, blood pressure, weight control, improved self-image, decreased stress, and decreased risk of coronary heart disease and stroke as well as other chronic diseases (125). Physical activity can reduce illness from chronic diseases and premature death and can lead to longer life expectancy (125). Physical activity also has mental and cognitive health benefits: it enhances brain plasticity and growth, as well as creative thinking and academic performance (126, 127).

Mindfulness has been defined as a state of present-moment awareness of one’s internal states and surroundings (128). Training in mindfulness involves learning to observe emotions, thoughts, and present moment experiences without judgment or reaction (128). Mindfulness training has been shown to alter or enhance specific aspects of attention by re-training basic cognitive processes from the default mode of constant distraction, also known as receptive attentional skills (129). In a space of mindful awareness, an individual experiencing harmful or distressing thoughts and emotions can view them as transient and accept them (130). Mindfulness can increase prosocial dispositions, including empathy and compassion, toward the self and others (131).

A recent study suggested several potential PPIs that could be implemented within veterinary education institutions (62). One of the proposed PPIs was to promote and encourage activities of daily living, including physical exercise (62). Several veterinary programs have sponsored yoga or other physical fitness programs on campus (62). Mindfulness interventions have also been explored in veterinary student and practicing veterinarian populations (59, 132). Findings include that regular mindfulness practice (once per week or more) was protective against anxiety and depression in veterinary students (133). In general, making healthy choices easy and social in nature make it more likely that healthy habits will be adopted (134). Facilitated peer support (132) and modeling of healthy behaviors (135) were found to be important for effective adoption of mindfulness and physical activity interventions in educational environments.

#### 5.1.2 Group-level (we) interventions

Group-level positive interventions refer to those tasks and strategies that involve individuals working on their wellbeing with at least one other person or a team with whom they are in regular contact (85). The literature explores various interventions at this level, including job crafting (29), building high-quality connections (136, 137), and group-level gratitude practices (84). Teamwork has been identified as the strongest retention factor for veterinary healthcare team members in a recent industry-wide survey (114). An emphasis on the team-based approach to veterinary healthcare is gaining momentum, with several recent publications disseminating data on the value of this approach (70, 138, 139, 140). The effectiveness of veterinary healthcare teams can significantly impact individual team member burnout and job satisfaction (141). Suggested interventions to enhance group and team-level wellbeing in veterinary education are presented in the following section, including animal-assisted interventions, integrating PERMA in educational environments, strengths-based coaching, positive psychology informed mentoring, experiential education, and cultivating connection.

##### 5.1.2.1 Animal-assisted interventions

The implementation of animal-assisted interventions (AAIs) on university campuses to decrease stress and improve wellbeing has become increasingly popular (142). AAIs on campuses typically involve therapy dogs, who can provide non-judgmental mental wellbeing support (142). Interacting with dogs has been shown to serve as a catalyst for social interaction, enhancing prosocial behavior and relationships that can contribute to overall wellbeing (143). Recent systematic reviews of AAIs in higher education show promising results in alleviating stress and anxiety (142, 144). The potential benefits of AAIs in veterinary education environments, including evaluation of the benefits of dog ownership, dog walking, and therapy animals for stress relief, has been presented (145, 146), and deserves further exploration. While AAIs have shown promise, it is crucial to ensure that such programs meet ethical criteria benefiting both animals and people. Collaborators in AAI should adhere to evidence-based practices that prioritize animal welfare to avoid introducing stressors or resistance in therapeutic interactions (147).

##### 5.1.2.2 Integrating PERMA in educational environments

The PERMA model (Positive Emotions, Engagement, Relationships, Meaning, and Accomplishment) can be integrated into educational environments to enhance both student and educator wellbeing (148). Weaving in the PERMA principles has been described to coalesce with existing evidence-based engaging learning strategies to promote and enable environments for flourishing in classroom settings (148). PERMA can be applied across various subjects and disciplines to guide the planning, implementation, and evaluation of learning objectives in the variety of contexts in which learning occurs (148). Veterinary pre-clinical and clinical education occurs in an array of environments, including both virtual and in person classrooms, laboratories, veterinary teaching hospitals, and work-based educational settings (6, 7), all of which offer opportunities for this type of integration. While PERMA is just one of many models used in positive education, it offers a framework that can be both implicitly and explicitly integrated into pedagogical/andragogical methods (148). This model has been shown to enhance learning, engagement, and overall wellbeing in students (149).

Positive emotions broaden thinking, increase engagement, and build enduring positive internal resources (11, 150). Educators can incorporate intentional elements into lectures, labs, rounds, and clinical rotations to enhance positive emotions and reduce stress. Implementation examples include storytelling (151), humor (152), evoking awe (153), incorporating savoring (108), and gratitude (148). A specific example would be to implement the “three good things” gratitude exercise into the learning environment, which has been shown to promote wellbeing, positive emotion, and supportive relationships (109).

Engagement in learning can be enhanced by integrating principles of self-determination theory, which posits that environmental nutrients essential for flourishing include autonomy, competence, and relatedness (154, 155). Practical examples include allowing students to select projects and research topics to foster autonomy, designing activities in such a way to develop competence according to their skill level, ensuring a balance between challenge and skill to enhance opportunities for flow (13), and utilizing peer learning and group work to enhance relatedness between classmates (148). An example that has been implemented in healthcare education is the integration of team-based learning in the early preclinical curriculum, which has been shown to increase engagement and improve communication skills (156), particularly in topics related to the basic sciences (157). Strengths-based coaching may also be a strategy to enhance engagement in the learning process (30).

Relationships are the most important element of the PERMA model with significant implications for flourishing and wellbeing (3, 158). High perceived social support is associated with improved wellbeing and positive health (159), and protects individuals from the negative impacts of stressful events (159). Relationships can be enhanced in educational settings by the incorporation of positive communication training (160), creating a supportive and psychologically safe environment (161), promoting teamwork through group activities and active debriefs (148), and providing respectful and supportive feedback (148). Positive relationships between students and educators can increase perceptions of belonging, which promotes academic success, mental health, and wellbeing (162).

Meaning can enhance belonging and can connect students and educators to a purpose for the learning that is bigger than the self (16, 122). Meaning can be enhanced by connecting the learning objectives to a real-world significance, being clear on the purpose of the learning, and encouraging reflection to evaluate comprehension of how the learning connects to broader contexts and goals (148). Leadership practices that have been found to enhance meaning at work that could be extrapolated to the educational context include highlighting the broader impact of the work, recognizing and nurturing potential, fostering personal connections, discussing values and organizational purpose, demonstrating integrity by modeling values-based behaviors, and granting autonomy (163). Examples of higher meaning and purpose in veterinary education include the importance of the human-animal bond (164) as well as the myriad ways in which veterinary professionals protect and promote public health and wellbeing (165). Meaning in veterinary work can be additionally enhanced and explored utilizing the job crafting exercise (166).

Accomplishment is related to self-efficacy, a concept that refers to a person’s positive belief in their abilities and expectations of performing successfully (167). Self-efficacy is closely related to behaviors, actions, and motivations (167). In the educational setting, self-efficacy can be most efficiently stimulated through experiences of educational achievement, which leads to a sense of mastery and accomplishment (148). Accomplishment can be enhanced in educational settings by setting achievable and realistic goals aligned with students’ current competencies, providing opportunities for mastery, and supporting student growth by providing opportunities to get out of their “comfort zone” through motivation and confidence-building activities (148). It is important to consider that normalizing a healthy balance in accomplishment through both failure and achievement is important, as veterinary professional students and practitioners have been shown to exhibit high levels of perfectionism, which may negatively impact their wellbeing and ability to cope with stress (115, 168, 169). Proposed reasons include the high emotional demands of clinical work, the cumulative effects of stress, and the potential desensitization arising from repeated exposure to distressing situations. Addressing this within the curriculum’s content and practices could involve integrating mindfulness, self-compassion, structured reflection, and additional evidence-based self-awareness and self-management techniques.

##### 5.1.2.3 Strengths-based coaching

Positive psychology provides the growing field of coaching with an evidence-based framework and a defined scope of practice (170). A recent review article defined strengths-based coaching (also known as positive psychology coaching) as a short to medium term collaborative and professional relationship between individuals or teams with a coach, aimed at the development of personal resources and strengths to enhance positive states, traits, and behaviors (171) as well as positive leadership and team dynamics (172). Positive psychology coaching emphasizes strengths, which is a process designed to identify and promote strengths development in an individual or a group, often using validated character strengths assessments (14, 173). Coaching has been utilized as a method to improve team communication training for veterinary students (174).

Research also highlights the importance of coaching for sustainable implementation of positive education efforts, with a focus on coaching leaders, teams, and champions combined with a positive and supportive culture (172). Positive psychology coaching models could be developed and integrated within veterinary academic institutions. Recent research in the veterinary space has demonstrated that positive leadership has a significant impact on veterinary workplace wellbeing, organizational commitment, turnover, and job satisfaction (175). Utilizing strengths-based coaching to improve positive leadership outcomes has been demonstrated (176). Coaching can also be utilized as an evidence-based communication skill to facilitate proactive solution-focused behaviors to promote mental health and the use of wellbeing competencies in groups and teams (177). Instructional coaching can also be utilized to enhance educator relationships and strengthen teaching practices (178).

##### 5.1.2.4 Positive-psychology informed mentoring

Recently published mentoring guidelines provided a comprehensive framework to support effective mentoring in the veterinary profession (179). Mentorship is defined as a two-way relationship where an individual invests personal knowledge, energy, and time to help another individual grow and develop professionally (179). Mentoring interventions have been shown to improve mental health and wellbeing outcomes in veterinary professionals (180). Positive psychology constructs, including hope, optimism, and resilience, have been shown to mediate the effects between mentoring and positive performance in a longitudinal study of a formal mentoring program (181). Mentoring has positive impacts for both the mentor and mentee (179). Mentorship programs have been evaluated in veterinary medicine, both within and outside of formal education settings (182). The intentional addition of positive psychology principles, such as character strengths and strengths-spotting (99) would be a novel and innovative approach to enhance wellbeing outcomes in formal mentoring programs in veterinary education.

##### 5.1.2.5 Experiential learning

Experiential learning is defined as contextually rich, critically reflective, contextual-specific, and pragmatically active educational experiences (183). Experiential education offers an additional intervention pathway to foster team building and enhance group-level wellbeing among veterinary professionals. A contemporary example is the Veterinary Leadership Experience (VLE), which is an experiential education program that integrates emotional intelligence, communication skills, resilience, and conflict management into its curriculum (184). Held in a nature-based, camp-like setting, the VLE encourages participants to step away from their daily routines, engage in reflective activities, and build strong team relationships through non-strenuous physical activities like low ropes courses as well as through play-based activities, which are designed to promote collaboration, challenge by choice, and to apply leadership and emotional intelligence concepts in real-time (184). A recent outcomes assessment study revealed a significant impact on key skills, including emotional intelligence and social awareness (184). Experiential learning opportunities built into the curriculum and academic workplace have the potential to have similar benefits. Further studies specifically on wellbeing outcomes for experiential veterinary education programs like the VLE are warranted.

##### 5.1.2.6 Cultivating positive relationships

Two PPIs that have been shown to enhance positive relationships are Active Constructive Responding and High-Quality Connections. Active Constructive Responding (ACR) is a communication technique that fosters positive interactions and reinforces supportive relationships within teams (103). ACR is a form of positive communication where an individual responds to another’s good news or positive experience in an enthusiastic and supportive manner, expressing a genuine interest to “hear more” (102). Sharing personal positive events with others was linked to an increase in daily positive emotions and wellbeing, surpassing the impact of the positive event itself and other daily occurrences (185). Wellbeing is also increased on behalf of the person responding to the positive event (185).

The implementation of ACR within groups can lead to several positive outcomes. ACR promotes open and supportive communication, improving problem-solving and collaboration among team members (102). Strengthened relationships result from consistently responding positively to each other’s successes, building trust and camaraderie, leading to a more cohesive team environment (102). A supportive team atmosphere created by ACR can also buffer the impact of work-related stressors, reducing the risk of burnout and improving overall mental health (185). Feeling valued and supported through positive interactions can enhance job satisfaction and motivation, leading to better performance and lower turnover rates (102).

High-quality connections (HQCs) are brief, one-on-one interactions that are positive for both the individuals involved and the structural aspects of their connection (137). HQCs are characterized by mutual respect, trust, and active engagement between individuals (137). These connections improve individual functioning, such as cognitive processing speed, memory, and immune system strength, and help employees recover from challenges and adapt to change (137). HQCs also facilitate individual development, foster psychological safety, increase cooperation, and enhance organizational processes, such as coordination. In essence, HQCs are crucial for developing strong, supportive relationships that contribute to a thriving work environment (136, 137).

There are many ways to cultivate HQCs in an educational environment. The specific elements that have been found to be most important include increasing awareness of others by understanding who they are and what they do, monitoring non-verbal communication to ensure it conveys warmth and acceptance, practicing perspective-taking to empathize with others’ experiences, and shaping behaviors to demonstrate care and support (137). HQCs can occur with task-enabling support by assisting colleagues with necessary information and emotional backing (137). Lastly, HQCs are enhanced by incorporating organizational practices that facilitate connections, such as team-building activities and playful interactions (137).

#### 5.1.3 Organizational-level (us) interventions

Organizational-level wellbeing interventions include tasks and strategies that have an impact on the entire educational department and/or institution, including wellbeing-related policies, frameworks, assessments (85). The literature is mounting in favor of the importance of organizational-level wellbeing initiatives as opposed to focusing mainly on individuals (186). A recent large study of over 46,000 workers across 233 organizations in the United Kingdom found that participants in individual-level wellbeing programs, such as mindfulness and resilience training, did not have improvement in wellbeing outcomes compared to non-participants (186). A combination approach was suggested to simultaneously enhance job resources while also mitigating job demands (186).

Recent work in veterinary medicine focuses on the importance of institutionalizing wellbeing as a core value and implementing systemic changes in organizations, including in veterinary educational environments (49, 113). This section will examine several positive psychology-based organizational-level approaches that enable flourishing: fostering healthy work cultures, embedded mental health professionals and wellbeing champions, chief wellness officers, appreciative inquiry, the arts and humanities in veterinary education, the positive built workplace environment, and policies that support wellbeing.

##### 5.1.3.1 Fostering healthy work cultures

One of the major challenges to wellbeing within veterinary education, and more broadly across the veterinary profession, is burnout (53, 63, 187). Based on over 40 years of research, the World Health Organization (WHO) recently defined burnout as follows: “a syndrome conceptualized as resulting from chronic workplace stress that has not been successfully managed… characterized by three dimensions: feelings of energy depletion of exhaustion; increased mental distance from one’s job, or feelings of negativity or cynicism related to one’s job; and reduced professional efficacy” (188). Burnout is a phenomenon specifically within the occupational context and should not be applied to describe experiences in other life areas or as a medical condition (189). Burnout is not an illness in and of itself but can predispose individuals to subsequent mental and physical health conditions (189).Workplace stress and burnout in veterinary medicine significantly impacts productivity, efficiency, quality of care, the wellbeing of practitioners, and also has significant economic impacts (53).

Individual-level approaches, such as teaching mindfulness skills, may be most effective at addressing one component of burnout (exhaustion), but leave untouched the other two important elements (189). These interventions can help individuals develop more effective strategies to productively respond to workplace stress, but do not improve the nature of the workplace itself in ways that effectively reduce distress (189). Focusing on the individual relies too heavily on the assumption that workplaces cannot or will not change their practices (189). Individuals in the veterinary profession are responsible for their wellbeing, but most burnout drivers are rooted in the work environment (49).

In a recent Merck Animal Health Veterinary Wellbeing Study, four factors emerged which defined healthy work cultures: strong sense of belonging to a team, high degree of trust in the organization, candid and open communication, and sufficient time to provide high-quality patient care (5). This survey highlighted the areas that were most important in clinical veterinary practice contexts; a similar study specifically in a veterinary academic context has yet to be completed, to the author’s knowledge. Additional areas important to fostering healthy work cultures in academic settings can be further explored in future studies, including meaning and purpose, mattering, psychological safety, and agency.

Meaning and purpose in veterinary work often comes from a deep sense of calling to help animals, which aligns with the concept of eudaimonia (190). A comprehensive framework for understanding the positive contributions to wellbeing derived from veterinary work, focusing on eudaimonic principles such as meaning and purpose, the human-animal bond and other forms of positive relationships, and personal growth, has been described (191). By focusing on these elements, the framework suggested that veterinary work can be a rich source of fulfillment and satisfaction which can intrinsically be used to help mitigate the stresses associated with the veterinary (191). Veterinary education institutions can leverage this eudaimonic framework to intentionally focus the institutional vision and values on the higher meaning and purpose of veterinary work and how that connects to collective wellbeing (114).

Mattering, the feeling of being valued (making a difference in the lives of others) and adding value (having significance in one’s community), leads to more supportive and effective organizational cultures, including those in higher education (192, 193). Mattering is a central aspect of psychological health and wellbeing (194). Research highlights that when people feel that they matter, it positively impacts their motivation and job satisfaction (195). Mattering has been shown to be a key factor in promoting wellbeing (196) and decreasing burnout in nurses (197). Feelings of not mattering are implicated in suicidal ideation and depression (194). Policies, procedures, and campus-based initiatives to increase mattering at the organizational level have recently been reviewed (Flett, Khan, and Su 2019).

Psychological safety is the belief that the workplace is safe for interpersonal risk-taking (161). Research on the subject has demonstrated that psychological safety allows employees “to feel safe at work in order to grow, learn, contribute, and perform effectively in a rapidly changing world” (161), p. 23. Analogous to the research on burnout mitigation and prevention, the literature on psychological safety points to the importance of positive leadership practices, workplace support, and work design (161). Psychological safety has been highlighted as one of the four essential pillars of how to lead thriving organizations in the veterinary profession (175). Psychological safety has been identified as a key element of healthy and safe working environments in veterinary education (198).

Agency, or the ability to positively influence one’s environment, is also applicable to the study of organizational-level wellbeing across myriad contexts, including higher education (199). Increasing individual and collective agency through involving employees in decision-making to enhance their job control and social support, while simultaneously reducing workplace stressors, explains the effectiveness of available combined burnout interventions (200). Student agency has been defined as the ability to take ownership over their learning and educational pathway and environment (201). Questions of how to enhance agentic behaviors within institutions have broader implications for the capability for positive progress and innovation (202).

##### 5.1.3.2 Embedded mental health professionals and wellbeing champions

The Association of American Veterinary Medical Colleges (AAVMC) formally recognizes the importance of veterinary wellbeing professionals embedded in academic institutions (73). Both licensed mental health professionals as well as veterinary educators are recognized as important in efforts to care for student wellbeing and to implement and sustain intentional wellbeing interventions in veterinary education environments (73). Current initiatives are underway to further explore the interprofessional roles and responsibilities of both embedded mental health professionals and veterinary professionals to improve wellbeing in veterinary healthcare teams (113).

In addition to embedded mental health professionals, formally integrating workplace wellbeing champions can significantly enhance and sustain comprehensive organizational workplace wellbeing interventions (203). Wellbeing champions are self-identified individuals who value health and wellbeing, have a genuine interest in supporting their colleagues, and want to promote grassroots approaches to wellbeing programs (203). Wellbeing champions raise awareness, participation, understanding, and overall success of workplace health and wellness programs (203). They are not necessarily trained mental health professionals, although in some contexts, they may have this type of professional and educational expertise. Wellbeing champions are trained in evidence-based strategies to support and enhance wellbeing within their workplace context (204). A commitment to obtaining and using evidence-based practices is also important to add as a criteria for being a wellbeing champion, in order to avoid unintentionally causing harm or being ineffective because individuals have not received proper training (203).

The crux of the function of a wellbeing champion is to engage colleagues in activities that promote wellbeing (203). Wellbeing champions may be responsible for collaboration, communication, and providing feedback to leadership and human resources regarding program implementation and effectiveness (204). Champions promote wellbeing activities and interventions through a variety of formats, including print, electronic, and in-person communication using readily available program resources, and are provided the autonomy to promote programs that may be of personal and professional interest within their target population (203). Wellbeing activities and interventions may include multiple domains of wellbeing, including physical activity, team building, social interaction and connection, and stress management (203). Wellbeing champions are positive influencers within the work environment and their presence can lead to stronger cultures of wellbeing (203).

A wellbeing champion program at the Mayo Clinic has been shown to increase awareness of wellbeing opportunities, a greater sense of support, and higher perceived health and wellbeing (205). A recent survey of Mayo Clinic employees with over 46,000 responses revealed that having wellbeing champions in the work unit, coupled with an organizational commitment to employee wellbeing, was associated with better employee engagement, job satisfaction, and perception of personal wellbeing (203).

##### 5.1.3.3 Chief wellness officers

A Chief Wellness Officer (CWO) is a newer leadership role to academic institutions and healthcare systems that has been developed to enhance population health and wellbeing (206). The CWO is responsible for developing and implementing strategic wellness initiatives aimed at promoting healthy behaviors and addressing mental health and wellbeing issues among students, faculty, and staff. This role involves identifying systemic issues contributing to burnout and job dissatisfaction, devising innovative solutions to these problems, and fostering a culture of wellbeing across the institution (206). The effectiveness of the CWO role has been demonstrated through improved health outcomes and significant returns on investment in wellbeing and wellness programs (206).

In an academic veterinary institution, a CWO-type position on the leadership team could significantly enhance the wellbeing of students, faculty, and staff by tailoring initiatives and interventions to the unique challenges and needs within the institution (206). If the university/educational institution already employs a CWO, this position would provide formal representation for wellbeing matters important to the veterinary program. This proposed role would involve the creation of a comprehensive wellbeing strategic plan that integrates evidence-based practices to promote individual and collective wellbeing. This position would need to collaborate with various departments at the veterinary college, including human resources, student services, and clinical operations, to ensure a cohesive approach to wellbeing. Additionally, this position would be tasked with monitoring the effectiveness of wellbeing programs through regular assessment. Formalizing wellbeing into a full-time leadership position signifies its importance to the people working in the organization and to the broader community, and provides continuity and a unified approach to individual, team, and organizational level wellbeing efforts (206).

##### 5.1.3.4 Appreciative inquiry

Appreciative Inquiry (AI) is a strengths-based, collaborative approach to organizational development and strategic planning that focuses on identifying and amplifying the positive aspects of an organization (31). Instead of concentrating on problems and deficits, AI engages stakeholders in a positive psychology-based process of discovering what is working well, and collectively envisioning a future where individual, team, and organizational strengths are maximized (31). This method typically involves four stages: Discover, Dream, Design, and Destiny, collectively known as the 4-D Cycle. During the Discovery phase, participants share stories of peak experiences and highlight core values and successful practices. The Dream phase encourages envisioning the best possible future, building on the identified strengths. In the Design phase, participants collaboratively plan and prioritize actions to achieve the envisioned future. Finally, the Destiny phase involves implementing and sustaining these plans to realize the desired outcomes (31).

AI has been widely used in various sectors, including business, education, and healthcare, due to its potential to foster innovation, enhance engagement, and improve overall organizational performance (31, 207). AI places a strong emphasis on social collaboration and appreciative systems, and seeks innovative ideas from employees via affirmation, appreciation, and dialog (31). The AI process focuses on sustainable positive changes, not just problem-based short-term solutions, and provides a flexible framework for discovering and utilizing personal and organizational strengths and resources to achieve goals (31). AI has been successfully utilized across several fields, including education, and has been found to be a proven method to generate cultural changes, building capacity, and for organizational development (208). One study in a healthcare setting utilized AI as a method to enhance manager communication to support nurses dealing with burnout (208).

A recent review article aimed to synthesize existing literature on AI across three disciplines: healthcare, higher education, and management (207). AI was found to have a positive impact across all levels: individuals, teams, and organizations (207). Limitations of AI that were discussed included an exclusive focus on positive aspects, which can overlook negative organizational dynamics that need improvement. Some studies addressed this by incorporating negative experiences into the AI process to draw out organizational core values and solutions-focused approaches (207).

##### 5.1.3.5 The arts and humanities in veterinary education

Another approach that is prime for application and research in the context of wellbeing in contemporary veterinary education is the integration of the arts and humanities into the curriculum and cultures of the institutions and organizations (209, 210). The humanities are broad, encompassing music, philosophy, arts, and literature (211). The humanities can contribute to human flourishing by providing psychologically rich and meaningful experiences as well as fostering essential knowledge, skills, and abilities for psychological and physical wellbeing (212). Westgate and Oishi (213) highlight the role of art, music, and literature in fostering direct affective benefits and indirect cognitive benefits, offering both enjoyable experiences and fostering social abilities, motivations, and emotion-regulation skills. These experiences are not just temporarily nourishing, they also impart lasting skills that can contribute to long-term wellbeing (213). In the positive humanities, or the branch of learning concerned with human culture in its relation to human flourishing, a “eudaimonic turn” is advocated to explicitly recognize and commit to flourishing as a practical aim and central theme of study (211).

The integration of the humanities into veterinary education represents an innovative and potentially transformative systemic approach to enhancing wellbeing in the profession (214). Veterinary medicine has been slow to embrace the humanities in the education of veterinary professionals and as relevant to the profession (214). In contrast, the humanities have been embraced in human medical education, with 94% of medical schools reporting core or elective humanities coursework to the American Association of Medical Colleges (AAMC) in 2018 (209). The AAMC recently released a report affirming the importance of integrating the arts and humanities into competency-based teaching and learning in medical education alongside guidance on the change in culture and research required to achieve effective integration (209, 215). The incorporation of the humanities into the veterinary curriculum has the potential to provide positive impacts on veterinary professional students as they prepare to embark into a unique and complex profession (214).

The traditional veterinary professional curriculum focuses on technical and clinical competencies that have been developed with respect to outcomes associated with success as a “Day One Veterinary Professional” (95). In adapting to societal influence and expectations, veterinary education institutions are shifting to address the complexities and challenges surrounding the provision of care to animals in modern society (216). These complexities and challenges include the value of animal life, the morality of animal euthanasia, and the socio-cultural and economic factors that impact the human-animal bond and the accessible provision of veterinary care (214). Incorporating the medical humanities has the potential to equip veterinary professional students with the skills to navigate these challenges even more thoughtfully and compassionately (214).

The four fundamental areas in veterinary practice that have been proposed to benefit from the inclusion of the medical humanities are the value of animal life, end of life and palliative care, the human-animal bond, and the human factor within veterinary medicine (214). These four areas are interrelated and contribute to the most challenging situations faced in veterinary practice, including justifiable versus unjustifiable animal euthanasia, animal welfare concerns, access to veterinary care, and veterinary care provider wellbeing (214). The American Association of Veterinary Medical Colleges (AAVMC) Competency-Based Veterinary Education (CBVE) model serves as a framework for modern veterinary curricular design and assessment (95). Included in this framework is the domain of professionalism and professional identity, which includes competencies related to ethics, self-reflection, and wellbeing (95), providing an available pathway for the integration of the humanities.

A specific example of the medical humanities is narrative medicine. Narrative medicine can be taught through narrative competency, which is defined as the ability to absorb, acknowledge, interpret, and act on the stories and difficulties of others (217). Training in narrative competence can improve knowledge, skills, and abilities in the areas of observation, empathy, listening, and self-reflection (217), as well as “narrative reciprocity” (218). Narrative reciprocity is viewed as the two-way flow of narrative engagement and connection, as opposed to the paternalistic medical paradigm of doctor (human or veterinary) to patient (218). Narrative medicine has been found to be an effective pedagogical tool and can modify participants’ attitudes, knowledge, and skills (217). Another example of narrative medicine is medical improv, which takes the basic skills necessary in improvisational theater and applies them to clinical scenarios (219). Improv focuses on self-awareness and reflection, alongside spontaneity and creativity (219). In a study of human medical students, a medical improv elective course led to improvements in wellbeing, professional development, and communication (219).

Other examples of the medical humanities include the integration of music and art. Listening to music and viewing art can reduce stress as experienced through improved mood regulation, achieving self-awareness, and expressing social awareness (215, 220). A randomized controlled trial in nursing students evaluated the use of music therapy and progressive muscle relaxation prior to exams, which found promising results in the reduction of stress and improved academic performance. Music can enhance cognitive abilities and empathetic understanding (220). Group musical activities and art appreciation sessions can foster teamwork and communication skills (220). The integration of the visual arts and other forms of creative expression have been explored in the contexts of veterinary anatomy and cytology instruction (221, 222). Songs have been utilized to help teach cardiopulmonary resuscitation (223).

Ethical competencies in the CBVE model can be explored within the realms of philosophy in the humanities (95). Practical wisdom, emphasizing the role of moral will and understanding the telos (purpose) of our actions has a strong connection to human flourishing (224). Veterinary medicine is a field where most people find a great deal of meaning in their work; often, it is described as a calling (191). A dark side to meaning has also been described, where ascribing one’s identity and contribution to the calling can lead to burnout and poor wellbeing (190). Meaning can also paradoxically lead to diminished wellbeing and health when healthy working conditions are not provided (225). Both moral skill (character strengths) and moral will (good character) need to be applied to effectively navigate a variety of challenging moral and ethical scenarios (224), both of which can be taught through philosophy.

Medical humanities programs can improve empathy (226). The veterinary profession demands not only clinical expertise, but also profound empathy and compassion toward both animals and their caretakers (227). Empathy contributes to enhanced patient satisfaction and increased adherence to medical recommendations (227, 228). Demonstration of empathy can have positive outcomes for animal caregivers and clients, including eased emotional concerns during financial discussions (229) and increased satisfaction with office visits (230). Despite the importance of empathy, it has been shown to decline during veterinary medical training (231). A recent meta-analysis evaluated the educational efficacy of medical humanities on the empathy of medical students and healthcare professionals, which found that theoretical education combined with practical education (i.e., real-life patient care encounters) were more effective than theoretical education alone to increase empathy (226).

Integrating the humanities may not provide universal benefits to all faculty, staff, and students in veterinary education. The diversity in individual preferences and engagement levels with the humanities activities necessitates a flexible and inclusive approach, including offering various options, respecting individual choices, and preserving optimal engagement through higher levels of autonomy (154). A reasonable approach that has the potential to be effective within these boundaries would be to integrate the concepts and pedagogical opportunities provided by the medical humanities into existing curriculum and courses (214). Additionally, veterinary educators and administrative leaders can embrace and culturally embed the medical humanities in other ways outside the classroom and clinic.

##### 5.1.3.6 The positive built workplace environment

An opportunity exists to explicitly link the constructs of positive psychology to the physical built workplace environment in educational institutions. A recent article explored this concept and introduced the Positive Built Workplace Environment (PBWE), which described how well-designed, sustainable contemporary workspaces can enhance employee performance, wellbeing, and organizational culture (232). Through a qualitative case study, the research highlighted the significant impact of a positive physical environment on job performance and employee engagement, along with fostering a sense of purpose and wellbeing among employees (232). The findings suggested that integrating positive psychology with workplace design can create environments that support sustainable high performance and wellbeing, advocating for further interdisciplinary research to enhance flourishing in workplace settings (232). Real-world design strategies to improve human and veterinary patient wellbeing within veterinary clinical and educational facilities were explored at a recent international veterinary education conference (233).

##### 5.1.3.7 Policies that support wellbeing

Best practices for policies to enhance wellbeing in organizations have been reviewed (234). Robust mental health benefits were found to be crucial, including access to a broad range of services (234).Workplace policies and practices to enhance opportunities for stress recovery, social connection, and access to healthy food and physical activity options were highlighted (234). Leadership buy-in, modeling, and support were found to be essential to sustainable policy implementation (234). Outcomes that indicated successful policies included increased awareness and utilization of wellbeing resources and initiatives, continuity of wellbeing implementation efforts, improved measures of health and wellbeing, and reduced stigma for help-seeking behaviors and toward mental health in general (234, 235). Additional policies that veterinary education institutions can consider include amending promotion and tenure metrics of success to incorporate efforts to enhance individual and collective wellbeing within the organization. Formal policies to increase work-related wellbeing and engagement by increasing resources and decreasing unreasonable job demands, such as re-thinking veterinary educator roles and responsibilities outside the traditional academic model of teaching, research, and service (236, 237), are also warranted. The importance of effective communication from leadership when it comes to making wellbeing related resources and policies understandable and widely available cannot be understated.

## 6 Measuring wellbeing in veterinary education

Wellbeing should be actively monitored and recognized as a key quality indicator and measurable outcome in all veterinary organizations, including in education (49). Evaluating wellbeing in educational settings highlights the importance and value of wellbeing to the institution (85). Measurement can guide the development of intervention programs by identifying areas of greatest need and impact, as well as to help to identify at-risk individuals and teams that may need additional resources and support (85). Selection of measurement tools will need to align with the educational institution’s context and values (85). Validated survey methods to assess wellbeing across the organization include the PERMA Workplace profiler (9), the Flourishing Scale (238), and the Culture of Mattering Survey in Higher Education (192). Additional measures that are currently being used in recent profession-wide wellbeing studies include the Kessler Psychological Distress Scale (239), The Mayo Clinic Physician Burnout and Wellbeing Scale (240), and the Big Five Personality Assessment (241).

Across institutions and contexts, assessment of wellbeing focuses on broadly defined psychological wellbeing (85). Assessment strategies depend on the wellbeing definitions and models chosen by the institution to use as frameworks (85). The factors impacting and driving wellbeing in the population of interest also need to be considered (85). A wellbeing assessment plan will need to evaluate the program’s effectiveness, impact, and sustainability (85). An evaluation strategy may include intended outcomes such as increased wellbeing awareness, improved wellbeing scores, decreased burnout, increased engagement in wellbeing-related curriculum and activities, and increased mattering (85, 242). Qualitative feedback can be collected through focus groups and interviews to gain in-depth insights and feedback. Participation and engagement metrics can track activity rates, and support and resources provided through wellbeing-related initiatives. Continuous improvement will need to be based on ongoing data analysis, outcomes assessments, and feedback (85).

## 7 Limitations and considerations

Implementing positive psychology interventions and approaches in veterinary education will require careful consideration of goals and resources. Potential challenges may include ensuring broad and equitable engagement and participation and aligning interventions with institutional goals and values. Common practical challenges to implementing positive education include both time and physical resources (85). Veterinary professional education programs are typically pressed for additional curriculum time, physical space, and resources. Ensuring that any proposed wellbeing initiatives align with competency-based veterinary educational frameworks and other objectives essential for achieving and maintaining institutional and programmatic accreditation are also significant considerations.

Additional measures will likely be needed to fully address the myriad upstream causes of wellbeing challenges in veterinary education and the broader veterinary profession. Extrapolation from the National Academy of Medicine’s National Plan for Health Workforce Wellbeing provided seven priority areas as a framework to inspire collective action to improve wellbeing in veterinary healthcare teams (113). Many of these seven priorities can be advanced using evidence provided by positive psychology, including those related to creating and sustaining positive work and learning environments and culture, investing in measurement, assessment, strategies, and research, supporting mental health and reducing stigma, and institutionalizing wellbeing as a long-term value (113).

The evidence-based systems-informed positive psychology approaches explored in this paper should not be considered an exhaustive list. Areas left to further explore that may lie outside the realms of positive psychology may include addressing compliance, regulatory, and policy barriers for daily work; engaging effective technology tools (including electronic health records and business models); and the recruitment and retention of a diverse and inclusive health workforce (113). Positive psychology does not offer clear solutions related to fair compensation and working in practices with modern medicine, two factors highly correlated with retention in the veterinary clinical workforce (AAHA (114)). An additional factor that deserves further research is workplace flexibility and schedule autonomy (243), as job flexibility was another strong retention driver in the veterinary professional workforce (70).

There will be the need to measure the effectiveness of interventions to evaluate outcomes using validated methods, which may present challenges associated with funding and other resources available to the institution (85). Ensuring accessibility, equity, and cultural humility in the personalization of applied positive psychology to suit diverse individual needs and contexts will be paramount (244). Assessment strategies need to carefully not over-value the onus of wellbeing on the individual; qualitative methods to gain insight about systemic factors affecting the workplace on individual, group, and organizational levels. Institutions may find that currently available validated assessment tools do not necessarily match their wellbeing goals, which may be another limiting factor.

## 8 Conclusion

Given the myriad concerns about veterinary mental health and wellbeing, the time is now for innovative upstream approaches to enhance wellbeing in the veterinary education space. Systems-informed applied positive psychology can be utilized to enhance efforts to promote flourishing in the veterinary education ecosystem. A change in the narrative is warranted to continue to ensure that the profession continues to attract and retain bright and optimistic professionals to the field across all the veterinary healthcare team and educator roles (49). Going above and beyond self-care and prioritizing a holistic and systemic approach to wellbeing in veterinary education will involve intentional and incremental changes to the structure and culture of the veterinary educational institutions themselves. New and developing veterinary colleges and institutions will have the advantage of creating such structures and cultures from the ground-up (245).

Further research will be needed on implementation and evaluation of PPIs and positive education specifically within veterinary student and veterinary educator populations to better understand how to measure competencies, assess outcomes, and evaluate return on investment for the educational organizations and institutions. Advances in technology and artificial intelligence will offer exciting frontiers for research and application. Enhancing the strengths of veterinary educational curricula, systems, and cultures with applied positive psychology has potential for significant positive impact, based on the growing fields of systems-informed positive psychology and positive education (35). Strengthening the wellbeing of veterinary professionals and veterinary education institutions enhances the quality of care provided to animals, which has myriad positive implications for animal caregivers, their communities, the environment, and society (5).
